# Abscisic Acid—Enemy or Savior in the Response of Cereals to Abiotic and Biotic Stresses?

**DOI:** 10.3390/ijms21134607

**Published:** 2020-06-29

**Authors:** Marta Gietler, Justyna Fidler, Mateusz Labudda, Małgorzata Nykiel

**Affiliations:** Department of Biochemistry and Microbiology, Institute of Biology, Warsaw University of Life Sciences-SGGW, 02-776 Warsaw, Poland; justyna_fidler@sggw.edu.pl (J.F.); mateusz_labudda@sggw.edu.pl (M.L.); malgorzata_nykiel@sggw.edu.pl (M.N.)

**Keywords:** abiotic stresses, abscisic acid (ABA), barley (*Hordeum vulgare* L.), biotic stresses, wheat (*Triticum aestivum* L.)

## Abstract

Abscisic acid (ABA) is well-known phytohormone involved in the control of plant natural developmental processes, as well as the stress response. Although in wheat (*Triticum aestivum* L.) and barley (*Hordeum vulgare* L.) its role in mechanism of the tolerance to most common abiotic stresses, such as drought, salinity, or extreme temperatures seems to be fairly well recognized, not many authors considered that changes in ABA content may also influence the sensitivity of cereals to adverse environmental factors, e.g., by accelerating senescence, lowering pollen fertility, and inducing seed dormancy. Moreover, recently, ABA has also been regarded as an element of the biotic stress response; however, its role is still highly unclear. Many studies connect the susceptibility to various diseases with increased concentration of this phytohormone. Therefore, in contrast to the original assumptions, the role of ABA in response to biotic and abiotic stress does not always have to be associated with survival mechanisms; on the contrary, in some cases, abscisic acid can be one of the factors that increases the susceptibility of plants to adverse biotic and abiotic environmental factors.

## 1. Introduction

Wheat (*Triticum aestivum* L.) and barley (*Hordeum vulgare* L.) are major cereal plants cultivated in temperate climate areas; however, various environmental stresses such as drought, salinity, extreme temperatures, and heavy metals, along with pathogens and pests, limit their yield and thus affect economic aspects of their agricultural production. Therefore, understanding the mechanisms of plant tolerance to stresses is of paramount importance. It is widely known that one of the plant hormones—abscisic acid (ABA)—is crucial for regulation of stress tolerance; however, its role is not limited to the response to abiotic stresses, as it is also involved in many developmental processes such as seed dormancy and germination or seedling growth [1,2]. Besides its well-established role in physiological processes and acclimation to abiotic stresses, ABA is recently considered as a regulator of biotic stress responses [3]. Many studies emphasize that ABA is a positive regulator of plant stress-resistance, although in some circumstances it can increase susceptibility to unfavorable environmental factors. In this review, we show the dual role of ABA in wheat and barley responses to abiotic and biotic stresses, which is an innovative approach. ABA is presently described mainly as a factor of resistance to adverse growth conditions, and its role in response to biotic stresses is often overlooked.

## 2. Abscisic Acid

### 2.1. ABA Metabolism

Modulation of ABA concentration in plant tissues is crucial for eliciting physiological responses, and it is associated with several simultaneously occurring processes: biosynthesis, catabolism and conjugation/deconjugation of this phytohormone.

**Figure 1:**
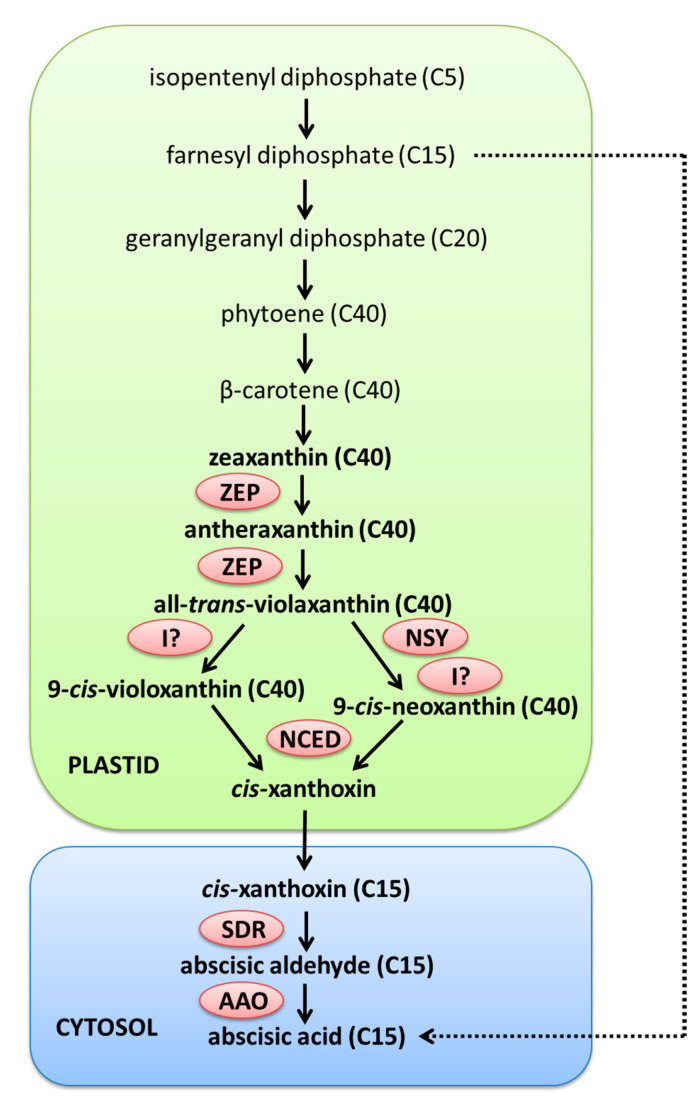
The biosynthesis of abscisic acid (ABA) in higher plants occurs via an indirect pathway (marked with solid, black arrows) and is initiated from the cleavage of β-carotene (C40). Initial steps in ABA biosynthesis occur in plastids, while final reactions take place in cytosol. In a direct pathway (marked with dashed, black arrow), which occurs in some fungi, ABA synthesis starts with the β-carotene precursor farnesyl diphosphate (C15). ZEP, zeaxanthin epoxidase; NSY, neoxanthin synthase; I?, unknown isomerase; NCED, 9-cis-epoxycarotenoid dioxygenase; SDR, short-chain alcohol dehydrogenase/reductase; AAO, abscisic aldehyde oxidase.

ABA is a 15-carbon (C15) sesquiterpenoid with two asymmetric centers, and due to the occurrence of one of them (at C-1’),two ABA forms are distinguished: natural (+)-ABA (*cis–trans*) and its unnatural stereoisomer (−)-ABA (*trans–trans*) [4]. ABA synthesis in higher plants occurs via a carotenoid pathway, which is also called an indirect pathway, because it is initiated from the cleavage of β-carotene, the C40 carotenoid precursor, contrary to a direct pathway which starts with intermediates containing 15 or less carbon atoms (Figure 1) [5,6]. Initial steps in ABA synthesis occur in plastids, while final reactions take place in cytosol. The first specific reaction in the ABA synthesis pathway is two-step conversion of zeaxanthin to all-*trans*-violaxanthin via antheraxanthin, which is catalyzed by a zeaxanthin epoxidase (ZEP) [7]. The *trans*-violaxanthin is then converted to 9-*cis*-violaxanthin and 9-*cis*-neoxanthin, but this stage is still not fully understood. Most likely, neoxanthin synthase (NSY) and an unknown isomerase are involved in the formation of *cis*-isomers of neoxanthin, while *cis* isomers of violaxanthin are formed directly in the reaction catalyzed by an unknown isomerase [8]. The last reaction of the ABA biosynthesis pathway, which occurs in chloroplasts, is catalyzed by a 9-*cis*-epoxycarotenoid dioxygenase (NCED) and is a rate-limiting step in ABA biosynthesis. NCED catalyzes the oxidative cleavage of 9-*cis*-violaxanthin and/or 9-*cis*-neoxantin to C15 *cis*-xanthoxin and C25 metabolite [9]. Although both 9-*cis*-violaxanthin and 9-*cis*-neoxantin can be used for ABA biosynthesis, it seems that the second one is the preferred substrate; when it is absent, insufficient ABA synthesis occurs to gain resistance to water deficit [8]. The last two enzymatic reactions of the ABA biosynthesis pathway take place in the cytosol to which *cis*-xanthoxin is transported from chloroplasts by an unknown mechanism [6]. The conversion of *cis*-xanthoxin to abscisic aldehyde is catalyzed by a short-chain alcohol dehydrogenase/reductase (SDR) [10]. Then abscisic aldehyde is oxidized to ABA in the reaction catalyzed by an abscisic aldehyde oxidase (AAO) [11].

To date, two alternative ABA catabolism pathways have been confirmed: hydroxylation and conjugation with glucose [6,12]. The dominant pathway for ABA inactivation is hydroxylation of the methyl group at the C-8’ position of the ABA ring structure, catalyzed by ABA 8’-hydroxylase (ABA8′-OH). As a result of this reaction, an 8′-hydroxy ABA derivative is formed, which is then spontaneously isomerized to phaseic acid (PA) [13,14]. In the next stage, PA is catalyzed by a PA reductase (PAR) to dihydrophaseic acid (DPA) and its 4’-O-B-glucoside (DPAG) by an unknown glycosyltransferase (GT). It is assumed that PA, DPA, and DPAG are inactive ABA metabolites, although some reports indicate that PA, in some physiological processes, acts as a hormone via the activation of ABA receptors [15]. Hydroxylation of ABA can also occur at the C-7’or C-9’ positions and results in a formation of derivatives of 7’-hydroxy-ABA and 9’-hydroxy-ABA, respectively. As a result of the spontaneous isomerization of the 9’-hydroxy-ABA derivative, neophaseic acid is formed (neoPA), while the 7’-hydroxy-ABA derivative is metabolized into an unknown product [12,16]. Interestingly, 7’-, 8’-, and 9’-hydroxy derivatives of abscisic acid exhibit biological activity, which was found to be higher than that of ABA in a number of studies. This indicates that these specific ABA metabolites may be involved in causing some or all of the ABA-mediated physiological effects [16,17].

The second ABA catabolic pathway is that of conjugation. The most common ABA conjugate, located in various organs in many plant species, is the ABA glucose ester (ABA-GE) [18,19]. Unlike oxidative inactivation of ABA, the formation of inactive glucose conjugates is a reversible process, and it is catalyzed by UDP-glucosyltransferase ABA (ABA UGT) [20,21,22]. The ABA glycosylation may play an important role in the transport of this phytohormone, enable its storage, and also provide protection against its degradation [18,20,23]. In addition, ABA-GE may undergo hydrolysis catalyzed by a β-glucosidase (BG) releasing biologically active ABA [24]. Hydrolysis of ABA-GE to ABA occurs in one step and, in comparison to ABA de novo biosynthesis, it can lead to a much faster increase in ABA concentration, which is particularly relevant in the response to changing environmental conditions [24,25].

The contribution of the cereal genes involved in ABA metabolism in response to various abiotic stresses could be verified based on either their overexpression or decreased expression (mutants, knockouts, RNAi approach) in transgenic plants (as summarized in Table 1).

### 2.2. ABA Signaling in Response to Stress

The last decade brought some findings concerning the mechanism of ABA signaling, which are in turn crucial for understanding the action of this phytohormone at the molecular level.

**Figure 2:**
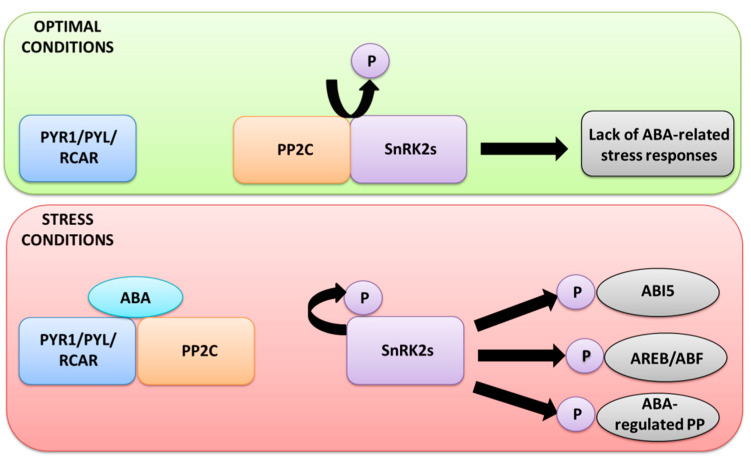
Changes in the ABA - receptor binding under optimal and stress conditions, and resulting alternations in ABA-dependent signal transduction pathways.PYR1/PYL/RCAR - protein receptors pyrabactin-resistance 1/pyrabactin resistance-like/regulatory component of ABA receptors; PP2C-protein phosphatase 2C; SnRK2-activates sucrose non-fermenting 1 (SNF1)-related protein kinases 2; ABI5-ABA INTENSIVE 5; AREB-ABA-responsive element (ABRE)-binding proteins; ABFs-ABRE-binding factors; PP-phosphoproteins.

ABA perception occurs when the phytohormone binds to the pyrabactin-resistance 1/pyrabactin-resistance-like/regulatory component of ABA receptors (PYR1/PYL/RCAR also referred to as PYLs) protein receptors [31,32], as shown in Figure 2. ABA receptor proteins contain a conserved START (steroidogenic acute regulatory-related lipid transfer) protein domain and thus belong to START protein superfamily [33]. PYLs are well described in *Arabidopsis*, in which the PYLs family contains 14 members [34]. Contrary to *Arabidopsis* PYLs, data about cereal ABA receptors are limited. In rice, 13 PYLs orthologues were identified; among them, 10 seem to be functional ABA receptors. Members of the rice PYLs family were differentially expressed in distinct tissues in response to ABA treatment, which suggest their specificity in the ABA signaling pathway and diverse biological function [35,36]. Binding of ABA to PYR1/PYL/RCAR receptors leads to inhibition of a protein phosphatase 2C (PP2C) activity, which in turn activates a sucrose nonfermenting 1 (SNF1)-related protein kinase 2 (SnRK2). Activated by autophosphorylation or other protein kinases, SnRK2 then phosphorylates relevant substrates (transcription factors and proteins), leading to ABA-related physiological responses [32,37]. In the absence of ABA, PP2Cs dephosphorylates SnRK2s, so the inhibition of their kinase activity occurs and the following steps of ABA signaling are consequentially precluded [38]. Recent molecular and genetic studies enabled identification of transcription factors playing a key role in the regulation of ABA-related gene expression under stress, including ABA-responsive element (ABRE)-binding proteins (AREBs), ABRE-binding factors (ABFs), and ABA insensitive 5 (ABI5) [39,40]. In response to stress condition, the ABA signal transduction cascade activates SnRK2s, which then regulate the activity of listed elements, as well as enzymes and phosphoproteins, which could be potential targets for SnRK2s [41].

Despite the significant progress that has been made in understanding ABA signaling and response at the molecular level, literature data on cereals are still limited. Recently, Tian et al. [36] reported group of PYL orthologs in rice (*OsPYL*) and discovered that the overexpression of *OsPYL3* and *OsPYL9* enhanced drought and cold stress tolerance. Similarly, the overexpression of wheat *TaPYL4* resulted in delayed wilting, improved grain production under drought, and increased water-use efficiency in transgenic plants compared to wild-type wheat [42]. As previously mentioned, PP2Cs are negative ABA signaling regulators, and plants with mutation in genes encoding these proteins exhibit ABA-hypersensitivity. Additionally, overexpression of maize *ZmPP2C* in *Arabidopsis thaliana* led to decreased tolerance to osmotic stress (induced by NaCl or mannitol) [43]. On the other hand, PP2Cs were induced by stress conditions in many species. The overexpression of maize *ZmPP2C2* in tobacco enhanced cold tolerance of transgenic plants by means of increased germination rate and higher activity of antioxidant enzymes, among others [44]. Similarly, expression of rice *OsPP2C* (*OsPP108*) was highly induced under osmotic and drought stresses, and its overexpression enhanced tolerance of these stresses [45]. It seems that a higher PP2C level can be considered as a part of the mechanism that desensitizes plants to high ABA levels [46]. Contrary to PP2C, SnRK2s are positive regulators of ABA signaling. Expression of ten identified wheat *TaSnRK2* genes was enhanced by water deficit and salt and cold stresses, where water stress (induced by PEG) had the greatest effect on increasing expression of *TaSnRK2s* [47]. Furthermore, overexpression of *TaSnRK2.4* in *A. thaliana* enhanced tolerance to drought, salt, and cold stresses. Transgenic plants exhibited decreased rate of water loss, improved membrane stability, and more robust photosynthetic capabilities compared to wild-type plants [48]. Similar results were obtained by Zhang et al. [49] for transgenic *A. thaliana* with overexpression of *TaSnRK2.8*.These results indicated that SnRK2s may play a key role in regulation of ABA-responsive genes via phosphorylation of AREBs, ABFs, or ABI5 in the response of cereals to various stresses. In addition, SnRK2 may also be essential to the activation of phosphoproteins important to the stress response, like the antioxidant enzymes ascorbate peroxidase (APX) or superoxide dismutase (SOD), as the use of proteomic approaches indicated that these enzymes are ABA-regulated in rice leaves [41].

### 2.3. Transport and Production of ABA Under Stress

Changes in ABA content are part of the adaptation of plants to dry environment. It was shown that drought-tolerant cultivars have higher ABA levels than the susceptible ones; however, the source of the ABA increase is still unclear. It was suggested that drought-stressed roots produce ABA, which is then transported to leaves via the xylem as a part of a systemic root-to-leaf drought signal, but Christmann et al. [50] proposed that this systemic signal could cause the ABA synthesis directly in leaves.

However, increase in ABA content in response to stress is not only dependent on de novo biosynthesis. ABA-GE (ABA glucose ester) is the main form of the accumulation of ABA in intracellular storage organelles and xylem sap, and it is probably the main form in cytosol and the cell wall as well [51]. In the drought-stressed barley roots, ABA-GE can be easily hydrolyzed by BG1, leading to an increase in the active ABA pool. Accumulation of ABA-GE was also observable in response to cold stress in wheat and barley [52]. BG synthesis was shown to be induced under osmotic and drought stress, leading to ABA release by ABA-GE cleavage [53]. Lee et al. [24] demonstrated that BG1 was located in the endoplasmic reticulum and remained there during stress responses. Interestingly, it seems that these findings are also applicable to the local synthesis of ABA in leaves. The rapid increase of ABA during stress was probably caused by the hydrolysis of a pre-existing pool of inactive ABA-GE and de novo biosynthesis, although which of these pathways is the leading one remains to be seen. Moreover, changes observed by Thameur et al. [54] in ABA and ABA-GE concentrations suggested that conjugates of ABA can play a key role in barley acclimation to drought conditions.

However, apart from the described “root-to-leaf” pathway, which is predominant in drought stress, changes in “leaf-to-root” stress response pathway were also observed in plants. Some stresses, such as heat, are primarily detected by leaves, which are exposed to different weather conditions. In response to increased temperature, wheat seedlings showed changes in the levels of stress-responsive gene expression, which was the effect of the suppression of ABA efflux from shoots to roots, and in phytohormone accumulation in leaves, which resulted in stomata closure [55].

Overaccumulation of ABA in cold-sensitive plants was associated with ZEP and NCED activation and inhibition of ABA 8′-hydroxylase (increased synthesis and decreased degradation). In stress-tolerant plants, ABA was maintained at the lower level, by inhibition of ABA synthesis enzymes (ZEP, NCED), and at a higher degradation rate [29], which was further proven by higher accumulation of ABA degradation products (PA and neoPA) in wheat and barley plants undergoing cold stress [52].

Moreover, besides changes in ABA content in response to stress, alternation of its activity may also be noticed. UV-B may lead to ABA inactivation by isomerization to 50% *cis–trans* and 50% *trans–trans*forms [56]. 

## 3. Role of ABA in Abiotic Stresses

### 3.1. Resistance of Wheat and Barley to Abiotic Stress

The most common abiotic stresses are drought, salinity, low and high temperature, heavy metals, and UV-B radiation. These stresses can severely affect wheat and barley production, causing drops in grain yield. 

Wheat, after maize and rice, is the third most cultivated crop worldwide. World wheat production in 2017–2018 was 730 million metric tons [57]. Heat stress, when combined with drought, is one of the major limitations to food production worldwide. What is cultivated due to its large yield tier, valuable nutrition properties, and unique technological characteristics of the grain [58]. Periodic water deficiency is one of the most important environmental factors limiting crop yields, including cereals (especially spring ones, which do not use winter soil water reserves) [59]. In 2018–2019, a decrease in wheat production by 15.5 million metric tons was observed compared to the previous year. A particular decrease in wheat production was observed in the European Union (reduction by 2.4 million metric tons) as well as in Russia (reduction by 3 million metric tons) and Australia (reduction by 1 million metric tons) [57]. Barley, despite having a much lower output than wheat, is a quite important cereal plant. World barley production in 2018–2019 was 137 million metric tons [57]. It is especially popular in countries with extreme climatic conditions due to its ability to grow in far northern or southern latitudes. Spring barley is characterized by modest climatic requirements and a short growing season. It can even be grown around the Arctic Circle, where it matures quickly under the polar day conditions. On the other hand, winter barley is sensitive to frost, but in turn it tolerates heat and drought [60]. Therefore, it can be grown in dry steppes and even semi-deserts. However, it grows best in areas with a mild climate (no frost) and on good soils. Barley is also among the main temperate-climate cereals that best adapts to water shortage, salinity, and other abiotic stresses [61]. Due to its greater drought resistance, the 2018–2019 decrease in production was only 4 million metric tons compared to the previous year [57]. Wheat is far more sensitive to heat, although this sensitivity is also dependent on the developmental stage. For instance, the threshold temperature of vegetative development is reported to be 20–30 °C, whereas that of reproductive growth is 15 °C. However, from approximately 20 days before anthesis to 10 days after anthesis, wheat can withstand a temperature of 31 °C without a decline in grain number [62].

The heavy metal tolerance is dependent on the type of ion applied and particular cultivar traits. Generally, barley is considered to be more tolerant than wheat. For instance, barley and wheat are similarly resistant to lead (Pb) and zinc (Zn), whereas barley is more resistant to cadmium (Cd) and copper (Cu). Moreover, Cd and Cu show stronger inhibitory effects on plant growth and development than Zn and Pb [63,64,65]. However, the possibility of growing tolerant crops on heavy-metal-contaminated agricultural soils leads to the accumulation of toxic particles in the food chain, which is undesirable [63].

### 3.2. Hormonal Plant Response to Abiotic Environmental Factors

One of the early plant responses to stressors is a change in hormonal balance [66]. The most commonly observed response of plants to a stress factor is a decrease in the levels of hormones that are associated with stimulation of plant growth (gibberellins, cytokinins, and sometimes indolylacetic acid) accompanied by an increase in the levels of hormones linked to inhibition of cell elongation growth or acceleration of tissue maturation and/or aging, i.e., ABA, ethylene, and jasmonic acid (JA) or its methyl ester (JA-Me) [66]. The level of ABA usually increases rapidly wherever there is a change in the water relations in the cells under the influence of stress factors such as drought, high temperature, and salinity [66].

However, some exceptions are known. For example, in barley seedlings, the increase of the temperature to 4 °C above optimal caused the ABA content to decrease in shoots and increase in roots within 20 min from stress application, in comparison to control plants [67]. In wheat, a similar response was noticed following a high dosage of cadmium (Cd). Concentration of Cd at the level of 1000 µM was accompanied with lowered ABA content, although 10 times lower dosage (100 µM Cd) caused a typical increase in the ABA content [68]. Interestingly, the response to UV-B overexposure was not always accompanied with an increase in ABA. In wheat cultivars, it seems that there was no clear correlation between ABA content and plant stress sensitivity; however, cultivars with higher tolerance to radiation tended to show either an increase or no change in ABA level in response to UV-B exposure, and cultivars with higher sensitivity to radiation showed rather a significant decrease in ABA concentration [69]. Chen et al. [70] also showed that UV-B exposure did not significantly change ABA content of germinating wheat seedlings. However, it has to be emphasized that UV-B may change not only the content of ABA, but also its form. Therefore, the active ABA pool may be negatively affected by radiation, even when total phytohormone content may be similar or elevated in comparison to the optimal conditions [56].

### 3.3. The Role of ABA in the Physiological Response of Plants to Abiotic Stress

The capacity of plants to withstand the stress depends on their age and phase of development as well as the duration and the intensity of stress [71,72]. During germination and seedling emergence, tolerance is determined based on percent survival; during later developmental stages, tolerance is usually determined by the reduction of plant growth and leaf-related parameters. Most studies indicate that plants are particularly susceptible to stress in the seedling phase and during the early stage of vegetative growth as compared to the germination phase, which, however, is delayed as a result of stress. Examples are found in barley [73], corn [74], wheat [75], and many other cereals. Studies conducted on five barley genotypes showed that drought leads to a reduction in the number of green leaves by up to 20% [76]. Moreover, correlations were also found between ABA content and leaf size. The greatest increase in ABA was recorded by the drought-resistant cultivar which, in turn, also exhibited the greatest decline in leaf area compared to the susceptible genotypes [76]. According to Poorter [77], genotypes with lower growth intensities had lower water requirements, and therefore they did not exhaust the limited water reserve in soil. Stress particularly often reduced shoot growth more than root growth [78]. It also reduced the number of florets per ear, increased pollen sterility, and affected the time of flowering and maturity of wheat [74] and barley [79]. The accumulation of ABA in barley roots under heat stress triggered short-term suppression of leaf elongation, which was most likely elevated by the restoration of water content in shoots [67]. Furthermore, exposure of wheat to heavy metal stress caused an ABA-related decrease in plant growth and development [80]. However, the opposite effect was observed in the case of UV-B stress, where the reaction mechanism of ABA also involved the inhibition of ethylene synthesis in plants. Ethylene is a phytohormone responsible for the development of leaves, flowers, and fruits, and it is also involved in the control of plant senescence [81]. Therefore, the inhibition of ethylene synthesis leads to enhanced plant growth [26].

ABA regulates the root growth and architecture under drought and salinity, as mentioned above; however, the effect of stress application on roots is dependent on many factors, such as the positions of branch roots, the angle formed with the parent root, and root length. Therefore, for example, salt had a strong inhibitory effect on lateral root growth, while primary roots were less sensitive to salt stress [82]. ABA has been shown to suppress lateral root growth in many studies [83,84]. A similar reaction was also observed in response to high radiation. In barley, UV-B exposure changed the ABA level, which presumably led to the transition from longitudinal to transverse growth of roots of stressed plant [85]. Thus, it is likely that this rhizogenesis process may represent an adaptive response to stresses, as demonstrated in recent genetic studies [86].

### 3.4. Impact of ABA on Seed Dormancy and Germination Under Stress

When environmental stress such as salinity or drought occurs during germination, ABA is produced in seeds [87], upregulating transcription factors such as AB13 and AB15 that stimulate genes encoding the osmotolerance proteins and inhibit germination [88,89]. Viviparous1 (Vp1) is another transcription factor involved in the ABA-dependent signaling cascade during seed development as well as abiotic stress responses in maize. The gene expression of Vp1 was found to be diminished in maize embryos cultured on ABA-free medium; however, it was enhanced under salt and osmotic stresses and upon treatment with exogenous ABA [90,91]. Cao et al. [91] also showed that the maize *Vp1* promoter was active not only in the embryo but also in the aleurone layer of the developing seed, and it was also detected in phloem cells of leaves, stems, and cobs. McKibbin et al. [92] demonstrated the presence of *Vp1* homologues in the wheat genome at evolutionally conserved chromosomal locations relative to *Vp1* in maize. These authors concluded that mis-splicing of wheat Vp1 transcripts promoted the susceptibility to preharvest sprouting, and they indicated that transgenic wheat embryos expressing *Avena fatua Vp1* had enhanced responsiveness to exogenous ABA administration and these transgenic seeds were characterized by resistance to preharvest sprouting [92]. Increased ABA level induced dormancy at seed maturation and prevented untimely germination, which promoted plant survival by adjusting vegetative development to seasonal changes in the environment [93]. However, this is not always a desirable phenomenon. For example, high temperature could lead to so-called thermodormancy which is induced by increased ABA level [94]. In *H. vulgare*, a specific role for *HvNCED1* and *HvNCED2* in the regulation of ABA synthesis during secondary dormancy has been suggested, while expression of *HvABA8’OH1* could be regarded as a key gene regulating primary dormancy. Seed development in cereals is characterized by two peaks of ABA accumulation that occur during the mid- and late-phases of seed maturation. The accumulation of ABA in the latter peak (which is mainly synthesized in the zygotic tissues) plays a key role in the induction and maintenance of seed dormancy [95]. Endogenous production of ABA in barley is placed in the endosperm, and during seed maturation it is the embryo that is responsible for activation of genes responsible for ABA biosynthesis [96]. ABA can also activate a residual G1 kinase, which becomes inactivated in the absence of ABA [97]. This can be the reason for rapid germination in case of the insufficient ABA content. However, still little is known about biosynthesis and catabolism of ABA and their regulation in different grain tissues. 

### 3.5. A Common ABA-Dependent Response to Abiotic Stress

Abiotic stresses show a large degree of parallelism in respect to the physiological, biochemical, molecular, and genetic effects on crops [98]. Plants generally share a universal response to salinity and drought stress, which reduce water potential in the soil. The deficit of water and osmotic potential change are the most common physiological mechanisms that cause growth reduction. In addition to the disturbed water distribution in plants due to the decreased availability of water in the soil as a result of reduced osmotic potential, the Na^+^ and Cl^−^ ions accompanying salinity also have a toxic effect on plants [99]. Moreover, salinity, drought, and heat stresses led to reduction in photosynthesis, transpiration, and other biochemical processes associated with plant growth, development, and productivity [100,101]. The biochemical pathways affected by stress result in reductions in yield amount and quality due to the decrease of wheat seed weight and seed number [102]. This drop in plant productivity is especially important in crops such as wheat and barley [80]. All abiotic stresses lead to cell dehydration, which is the main source of the physiological changes in the plant organism [103]. Prolonged stress results in an imbalance between reactive oxygen species (ROS) formation rate and activity of the antioxidant system. This imbalance may be stronger in particular stresses, such as heavy metal presence in soil. Based on the redox reactivity, bioactive metals are divided into two groups: redox metals (e.g., Cr, Cu, Fe) and non-redox metals (e.g., Cd, Ni, Hg). Metals from the first group can directly lead to ROS overproduction via Haber–Weiss and Fenton reactions [104]. Non-redox metals mainly deactivate the cell antioxidative system by depletion of glutathione, the binding of protein sulfhydryl groups, and the inhibition of antioxidative enzymes [105]. ROS contribute to overall cellular damage resulting from oxidation-dependent deactivation of some proteins and damage of macromolecules such as lipids, photosynthetic pigments, and nucleic acids [106]. Even a slight reduction of water potential in roots or leaves by stress factors stimulates the de novo synthesis of ABA and triggers its release from the inactive bound form. 

ABA can flow by xylem from roots to leaves or from chloroplasts to cytosol and further—through cell walls to guard cells [103]. A reaction to ABA may occur in a very short time (e.g., closing of stomata as a result of changes in the transport of certain ions to guard cells induced by ABA) or requires a longer time for its realization (e.g., various metabolic responses, including changes in photosynthetic carbon metabolism).

ABA induces stomata closing by triggering a transient rise in cytosolic Ca^2+^ that in turn inhibits plasma membrane proton pumps and inward K^+^ channels and also activates anion channels, leading to the release of anions from the guard cells. Anion-efflux-induced depolarization activates outward K^+^ channels and leads to K^+^ efflux as well (reviewed in [107,108,109]). Reduced osmolarity in guard cells thus leads to water efflux and stomata closure. This is a common response not only to dehydration, but also to other stresses, such as heat. 

For further reduction of the water loss in the response to various stresses, physical barriers, such as waxes, occur in plants, and their production is influenced by ABA. In barley, accumulation of ABA in response to cadmium also led to upregulation of expression of genes encoding lipid transporter proteins (LTPs) [110]. LTPs are members of a protein family found in all land plants, whose main function in vivo is probably synthesis of lipid barrier polymers, such as cuticular waxes, suberin, and sporopollenin [111]. Therefore, ABA leads to increased wax synthesis, which further influences water status in crops. It was also found that the epicuticular wax layer might play a key role in drought tolerance of wheat and barley genotypes. Plants with thicker epicuticular wax layer on leaves showed a reduced loss of water from the plant leaf surface. Therefore, reduction of residual transpiration rate was associated with the drought tolerance in crop plants and has been used as a selection criterion in wheat and barley breeding programs [112].

ABA also strengthened physical barriers, protecting plant cells against the accumulation of toxic ions and xenobiotics. For example, in wheat, an increase in ABA content in response to heavy metal stress improved phenylalanine ammonialyase (PAL) enzyme activity, which caused acceleration of lignin deposition. Lignification of cell walls led to a decrease in cadmium ion penetration into the plants due to the strengthened physical barrier [113]. The same response was observed to counteract the toxic effect of salinity in barley and wheat. Highly increased activities of PAL and cinnamyl alcohol dehydrogenase (CAD), the latter being an enzyme responsible for lignin biosynthesis, in salinity-resistant barley clearly suggested their involvement in the tolerance mechanisms. In the tolerant wheat cultivar, a similar reaction was observed, but it was not as strong as that noticed in the resistant cultivar of barley [114].

There is ample evidence of ABA involvement in the acclimatization reactions of plenty of plants. As previously mentioned, ABA is a hormone that improves water management of the plant: on the one hand, it closes stomata and enhances wax production, which reduces transpiration; on the other hand, it can contribute to more efficient intake and conduction of water [115]. In wheat, distribution of ABA between the root and the shoot affected crop response to heat. Kudoyarova et al. [55] proved that wheat seedlings show changes in the level of transpiration in response to increased temperature (which was the effect of the suppression of ABA efflux from shoots to roots) and phytohormone accumulation in the leaves that resulted in stomata closure (which lowered transpiration). However, changes in water balance may be also observed in stresses not directly correlated with problems with water intake, such as heavy metal stress. Stomata closure, reduced water uptake, and inhibition of chlorophyll synthesis and photosynthesis were observed in response to cadmium. These responses led to a reduction in transpiration and, consequentially, to a reduction in water uptake by roots, which was correlated with lowering Cd transport into plants. Therefore, in response to stress, there was a noticeable decrease in plant growth and development, which was associated with accumulation of ABA [80]. 

Although the ABA increase in abiotic-stress-treated plants usually activates parallel, conservative acclimation pathways across cereals, some of the plant reactions are dependent on the type of stress applied. For instance, in response to heat, intensity of transpiration was still increased in plants grown in high temperature, but accumulation of ABA in roots allowed the improvement of hydraulic conductivity during air warming by changes in the activity of aquaporins [116]. This allowed plants to maintain the high level of transpiration without the reduction of water content in leaf, in addition to enabling resumption of elongation. Such a reaction was not observed in an ABA-deficient mutant [67]. ABA was also of paramount importance in the alarm-phase response of wheat to cold, where it enabled stabilization of water relationships by stomata closure and expression of frost-tolerance-associated genes such as *Wcs120* (gene encoding one of late embryogenesis abundant (LEA) II dehidrin-like proteins) [117]. 

To further evaluate the deleterious consequences of water imbalance resulting from various stress factors, several other ABA-mediated changes that are observed in cereals should be described. It is known that an increase in osmotic pressure in cells can be an important element of the mechanism enabling cell growth under conditions of water stress (the condition of so-called osmotic adjustment). To counteract this process, ABA takes part in the regulation of the synthesis of osmotically active metabolites such as proline, polyols, or soluble sugars [76]. For instance, under heat stress, ABA affects both galactinol synthase and β-amylase activities. These enzymes are involved in an increased accumulation of soluble sugars which act as osmoprotectants [118]. Similar responses of barley and wheat were observed in other stresses such as drought [76]. Moreover, in wheat seedlings undergoing Cd stress, it was also shown that the application of exogenous ABA led to an increase abundance of free proline and phenolic compounds; by acting as osmoprotectants, these compounds may partially alleviate the water stress emitted by reducing the uptake of water by plant roots [119]. However, an atypical response was observed by Ci et al. [120] while researching Cd stress, where heavy metal application led to a decrease in the total soluble sugar concentration in wheat. 

### 3.6. Role of ABA in Protein Expression under Different Stresses

In plant cells, the accumulation of ABA is related to oxidative stress. Although ABA may stimulate the production of H_2_O_2_ [121], it also induces the expression of selected antioxidant genes encoding, for example, catalase (CAT), peroxidase (POX) [121,122,123], glutathione peroxidase (GPX) [124], glutathione S-transferases (GST) [125], SOD, APX, and glutathione reductase (GR) [121], thus decreasing redox imbalance.

Research conducted by Szypulska et al. [126] on barley caryopses, which were pretreated with ABA (100 µM) before induction of salinity, showed the activation of defense mechanisms which could prevent the oxidative damage to lipid membranes and proteins. Strong induction of two major proteins, namely actin and putative formin-like protein, was shown. This may suggest the influence of ABA influence the organization of actin filaments. The cytoskeleton is involved in signaling, therefore the potential significance of ABA-induced actin reorganization should be recognized. Research by Szypulska and Weidner [127] revealed that ABA treatment increased the concentration of cytomatrix-bound polysomes, a fraction of polysomes that are attached to the membrane and the cytoskeleton, mainly actin microfilaments of triticale. They suggested the role of cytomatrix-bound polysomes in LEA protein synthesis. Their study also demonstrated the role of ABA in jasmonate-induced protein regulation, which is implicated in mRNA stabilization. It also has been shown that stress induces alterations in elongation factor 1α (EF1a) and histone acetyltransferase 2 (HAC2), which could point to ABA participation in the control of phosphatidylinositol signaling and chromatin-mediated mechanism of stress tolerance. The number of proteins responsive to desiccation stress was altered by ABA pretreatment, which further confirms that ABA contributes to protection against dehydration [126].

Moreover, in barley, heat stress resulted in overexpression of ABA-responsive transcription factor HvDRF1 (DREB2A-homolog) which, in co-operation with other ABA-responsive factors, led to upregulation of stress-related gene expression through the ABA-dependent pathway [128]. There was also a high level of the ABA-responsive element 1 transcription factor HvABF1, which interacts with 14-3-3 family proteins that function as the regulators of plant primary metabolism and ion homeostasis [129]. Furthermore, in response to heat, there was overexpression of the ABA-induced LEA gene *HVA22*, which has a potential role in heat stress response [130]. The main function of LEA proteins is to sequester ions accumulating in the cell and retain water molecules to avoid protein aggregation and enzyme deactivation.

LEA proteins, produced in an ABA-dependent manner, also have a significant role in osmotic tolerance under stress. For example, ABI5 transcription factor activates transcription of LEA proteins that act as osmoprotectants against cellular dehydration during drought [131]. Reduction of water uptake leads to plant dehydration, triggering an ABA-dependent response that includes accumulation of dehydrins, proteins whose main function is protection of cell structures against damage. Dehydrins are stress proteins with a high number of charged amino acids that belong to the Group II LEA family. ABA generates those dehydrins with ABRE promoters. For example, in root tips of barley, cadmium led to upregulation of the expression of dehydrin genes *DHN1* and *DHN6* [132], and in wheat it increased the expression of dehydrin gene *TaDHN* [133] and genes encoding dehydrin, namely *wzy1-2*. Dehydrins have many functions in alleviating stress, including participation in binding and detoxification of heavy metal ions, reduction of ROS accumulation [134], protection of biopolymers from denaturation, stabilization of cell membranes, and preservation of cell structure integrity under stress [135]. 

In response to cold, an enhanced level of ABA also led to higher abundance of not only proteins involved in stress defense, such as heat shock protein 70 kDa, WCS120, thioredoxin-dependent peroxidase, and thaumatin-like protein, but also those involved in protein folding, e.g., ribulose bisphosphate carboxylase/oxygenase (RuBisCO) large subunit-binding proteins and luminal binding proteins [52]. It seems that the WCS120 protein is of paramount importance in cold-response. ABA regulates the expression of the promoter of the wheat *Wcs120* gene, as well as its barley homologue *Dhn5*, which encode an important group of cold-inducible dehydrins. The abundance of WCS120 and its homologues in cold-acclimated plants has been correlated with the level of acquired frost tolerance. The WCS120 protein (and its homologues) can be regarded as markers of acquired frost tolerance in the cold-acclimated cereals [117]. 

Another important family of proteins involved in stress tolerance of plants, including wheat and barley, are the heat shock proteins (HSP) mentioned above. They are accumulated in response to numerous stresses, suggesting analogous response mechanisms. A close association between the HSP and ROS also exists, which suggests that plants use a controlled ROS level as elicitor to generate HSP for better adaptation through activating an array of molecules [136]. They are also present during some developmental stages. Abscisic acid concentration is known to be mainly increased in embryos; it is also enhanced in the endosperm during grain filling and maturation, which appears to cause HSP accumulation during these stages [137]. However plants grown at higher than optimal temperature accumulate substantially higher ABA and HSP levels in grains [138]. Heat shock factors (Hsfs) are central regulators in heat acclimation. In response to heat in transgenic wheat plants with overproduction of ABA, a high level of *TaHsfC2a* was observed, which led to upregulation of a suite of heat-protection genes, such as *TaHSP16.9b*, *TaHSP17.3*, *TaHSP26.6*, *TaHSP62.4*, *TaHSP70d*, *HSP101b*, *TaGalSyn*, *TaHSA32*, *TaRof1*, and *TaβAmy1* [118], which corresponded with higher levels of not only heat shock proteins [139] but also galactinol synthase [140] and β-amylase [141], which, in addition to other roles, are responsible for osmoprotection. Enhanced expression of *TaHsfC2a-B* was observed in the response of wheat to different stresses, including drought and heat; however, it seems that this gene only improved thermotolerance [118].

In addition, in studies conducted by applying exogenous ABA to drought-treated plants, increased contents of ascorbic acid (ASA) and glutathione (GSH) [142,143] and decreased malondialdehyde (MDA) and H_2_O_2_ contents [144] were observed in wheat seedlings. This suggests that exogenous application of ABA enhances the tolerance of wheat seedlings to water shortage, which is a similar finding to those in previous reports concerning maize, Bermuda grass, and grapevine [145]. According Wei et al. [144], ABA may temporally regulate the transcriptional levels of genes encoding ASA–GSH cycle enzymes in wheat seedlings, resulting in increased GSH and ASA contents. GSH and ASA are major nonenzymatic antioxidants, and together with the whole ASA–GSH cycle they play an important role in scavenging of ROS [146]. It was observed that GSH and ASA contents in the abiotic-stress-tolerant plant varieties were significantly higher than those in abiotic-stress-sensitive ones [147]. Overexpression of genes encoding ASA–GSH cycle enzymes in higher plants resulted in increased tolerance to abiotic stress (e.g., salt, low temperature) in wheat by maintaining higher GSH and ASA contents [148]. Similarly, high glutathione content and increased activity of ASA–GSH cycle seemed to be some of the factors responsible for drought tolerance in wheat seedlings [149].

In response to UV-B radiation, ABA increased nicotinamide adenine dinucleotide phosphate oxidase (pNOX) activity and H_2_O_2_ and NO generation, which on the one hand enhanced oxidative damage but on the other hand was responsible for activation of the plant acclimation mechanism. At the same time ABA increased the content of phenols, which act as a shield against radiation and ROS scavengers, giving a photoprotective effect. It is likely that those changes are highly beneficial for the plant, which explains why ABA biosynthesis mutants showed far greater leaf injuries in response to UV-B than wild-type plants [150,151].

### 3.7. ABA Regulation in Senescence

ABA is also involved in the control of developmental senescence by inhibition of stomata closure, which leads to acceleration of water loss in senescing leaves. Transcriptomic analysis of barley flag leaves indicated upregulation of genes encoding NCED and two ABRE proteins: a cytokinin oxidase (involved in cytokinin degradation) and an ACC oxidase (involved in ethylene biosynthesis) [152]. However some stresses, such as UV-B, may lead also to ABA-dependent ethylene synthesis inhibition [26]. Barley and wheat senescence are favored by low cytokinin concentration but are interfered with by enhanced abscisic acid and ethylene levels and by ABA-dependent signaling [152]. Furthermore, Zhang and Gan [153] have recently demonstrated in *Arabidopsis* that stomatal movement and water loss during senescence are controlled through a regulatory chain consisting of ABA, AtNAP transcription factor, and PP2C, suggesting a mechanism through which ABA is involved in the regulation of developmental leaf senescence.

Drought also accelerates the aging process, specifically through oxidative modification of chloroplast proteins leading to their degradation [154]. The same reaction was noticed in response to high temperature [155], cold [156], heavy metals [157,158], and UV-B [69]. Proteins involved in all steps of photosynthesis, as well as the assembly of the photosynthetic apparatus, were degraded, leading to reduced stress tolerance [159,160].

In barley, exogenous ABA application reduced chlorophyll content and affected the transcription of nuclear and chloroplastic genes encoding proteins involved in photosynthesis and processes related to it [161]. Many studies also reported that *Arabidopsis* mutants with deficiencies in ABA biosynthesis or signaling exhibit altered or delayed senescence [162,163].

### 3.8. Deleterious Consequences of ABA Accumulation

Although it seems that ABA is crucial for plant survival under conditions of abiotic stress, it also leads to some changes that may be considered as negative for the plant growth and development, and consequentially to yield, which is the main reason for which wheat and barley are grown.

In high temperature, not only plant growth but also seeds dormancy is affected. In barley, incubation of grains at 30 °C resulted in induction of secondary dormancy, which reinforced seed sensitivity to temperature and reduced germination ability. Secondary dormancy inhibited grain germination even after transfer to optimal conditions (15–20 °C). This phenomenon is associated with a high level of ABA and increased embryo sensitivity to this phytohormone and can be partially removed or reduced by an inhibitor of ABA synthesis (fluridone, 0.1 mM) [94]. Moreover, ABA-mediated expression of heat shock factor TaHsfC2a-B, although improving thermotolerance, was also associated with reduction in plant growth [118]. In addition, ABA could also act as a inhibitor of starch accumulation, causing reduction in grain mass [164]. These downsides of the ABA-related response to heat result in decreased crop yield, crucially impacting wheat and barley production.

ABA also manipulates reproductive processes, especially in low temperature. Cold can lead to flower abscission, pollen sterility, pollen tube distortion, ovule abortion, and reduction in grain set, all of which lower barley and wheat yield [79]. In wheat it was observed that accumulation of endogenous ABA in anther tissue was linked to male infertility [165]. Cultivars with lower stress sensitivity also showed lower levels of anther ABA and higher grain number. Furthermore, in response to cold stress, ABA is a probable factor in apoplastic sugar transport regulation in anthers. Repression of the anther CWIN gene, *TaIVR1* or *TaINV4* in wheat, led to a decrease in reduced free sugar concentration. Sugars are of primary importance in the protection against cold due to their role in membrane stabilization, OH^-^ scavenging, and participation in signal transduction, as well as their cross-talk between other hormone-dependent pathways. ABA accumulation in anthers leads to pollen sterility and flower abortion [166], which negatively influence crop yield.

Finally, it was observed that, in order to minimize Cd buildup in plants, accumulation of ABA under heavy metal stress led to dehydration of plants, which in turn negatively impacted wheat and barley growth and development [167]. Pretreatment of wheat seedlings with salicylic acid (SA) reduced the increase of ABA under cadmium treatment, which was associated with maintaining growth characteristics of wheat seedlings at the level close to the control, despite stress conditions, and acceleration of recovery mechanisms. Moreover, decline in amplitude of ABA accumulation was also associated with lower cell oxidative damage (measured in MDA content) and electrolyte leakage [113]. Therefore, cadmium-induced overaccumulation of ABA in wheat may in fact act as an aggravating factor for some of the negative effects of heavy metal stress.

**Figure 3:**
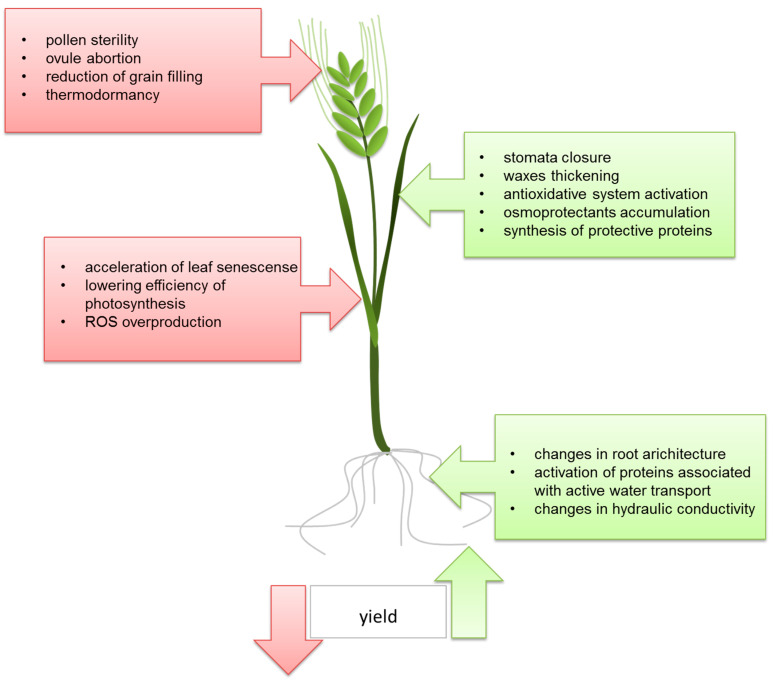
Overview of common ABA-related responses of cereals to abiotic stresses.

A brief summary of the common physiological changes, including both positive and negative factors, in the response of cereals to abiotic stresses is presented in Figure 3.

## 4. ABA in Cereals Responses to Biotic Stresses

### 4.1. Susceptibility of Wheat and Barley to Biotic Stresses

Pests and pathogens are major factors limiting the yield of crops, including wheat and barley [168]. There are around 200 documented pathogens and pests of wheat, and approximately 50 of them are considered significant in major wheat-growing regions of the world. Overall, pathogens and pests cause a 13% yield loss in wheat [169]. Among them, the most common are fungal diseases; they cause the highest damage to wheat, thus hindering wheat production. It is hard to achieve resistance to certain pathogens because, like in the case of rust pathogens (*Puccinia* sp.) and powdery mildews (*Blumeria graminis*), new races of these phytopathogens with new degrees of virulence continue to evolve, thus rendering resistance genes introduced through breeding ineffective [169]. Therefore, the majority of cultivars are still susceptible to the dominant pathogens. Moreover, there is limited genetic diversity of resistance genes available for use in breeding programs. The monoculture of modern wheat cultivars with low genetic diversity has resulted in pathogen resurgences, threatening wheat supplies [170]. Therefore, understanding mechanisms of biotic stress responses in plants is of paramount importance in the current situation.

As previously mentioned, barley, in contrast to wheat, can be grown in more extreme environments due to greater resistance to abiotic factors, and therefore it is endangered by the increasing range of phytopathogens. It is estimated that cultivated barley is a host to more than 250 different pathogens, and the most dominant are powdery mildew; head blight (*Fusarium* sp.); barley rusts; viruses such as barley yellow dwarf virus (BYDV); and pests, particularly aphids, beetles, and nematodes. The disease incidence depends directly on a few factors, including local climate, geography, soil type, prevailing agricultural practices, and plant age [171]. To minimize yield loss resulting from biotic stress, various disease-resistant varieties were created by introduction of resistance genes; however, due to the ever-present “arms race” between plants and pathogens and the resulting evolution of pathogens, this approach has proved to be ineffective in long term in many cases, such as that of wheat [172]. Therefore, in order to get a solution that works in the long run, more integrated molecular approaches for developing high-yielding barley varieties with enhanced durability and broad-spectrum disease resistance have to be applied [171].

In the light of this information, understanding the unclear role of ABA in plant responses to biotic stress can help to reduce yield losses resulting from diseases and infestations. As was described in previous sections, ABA is a well-known phytohormonal molecule that is involved in signaling during abiotic stress responses in plants, but the knowledge about its participation in wheat and barley responses against pests and pathogens is still fragmentary. Most research in this area has been conducted on noncereal species, such as *Arabidopsis* [173,174,175,176,177], Solanaceae species [178,179,180,181,182] or soybean [183,184,185,186,187]. Therefore, the summary of results showing the roles of ABA in wheat and barley responses to biotic factors is important because it can adumbrate future research paths that would be worth following. Available research suggests that ABA can modulate plant host responses positively or negatively depending on the pathosystem [188].

### 4.2. ABA in Fungual Diseases

It is clearly visible that in most of the described cases, ABA acts as disease susceptibility factor in wheat and barley plants, especially in relation to pathogenic fungi [188]. In general, pathogenic fungi can be roughly classified into two categories—biotrophic and necrotrophic—on the basis of their lifestyle; however, deviations from this rule are sometimes observed [170]. It seems that that the impact of ABA on the resistance of cereals to fungus is dependent on a few factors, like the intensity of increase of this phytohormone, the type of pathogen, the source of ABA (endogenous or exogenous), or the method of application. It is hard to point at a particular mechanism of an ABA-mediated answer in biotic stresses; however, it appears that abscisic acid may impact pathogen penetration and spread, as well as expression of genes encoding proteins associated with plant response to biotic stress.

One of the commonly used methods in phytopathological studies regarding the effect of various phytohormones on plants infected with pathogens is to spray leaves with solutions containing plant hormones. Ulferts et al. [189] reported that an ABA solution sprayed onto leaves of 7-day-old barley plants prior to inoculation with *Magnaporthe oryzae*, a hemi-biotrophic pathogen, caused more frequent and larger disease symptoms on ABA-treated leaves. Furthermore, microscopic observations revealed that increased barley susceptibility resulted from decreased penetration resistance. These authors also showed lower susceptibility to infestation by *M. oryzae* and enhanced penetration resistance in a barley mutant with disturbed ABA synthesis in comparison to the isogenic wild-type plants. Surprisingly, the endogenous amount of ABA was not significantly altered after *M. oryzae* attack [189]. For comparison to infection with hemi-biotrophic *M. oryzae*, the opposite results were presented by Wiese et al. [190] for barley plants infected with an obligate biotrophic fungus, namely *Blumeria graminis* f. sp. *hordei*. However, the other factor differentiating those studies was the ABA application method, as the roots were treated with ABA in nutrient solution in the study of Wiese et al. [190]. It has been shown that the pustule number of *B*. *graminis* on barley leaves was significantly restricted by more than 50% in an ABA-amount-dependent manner in nutrient solution, and the spread of *B*. *graminis* was controlled by formed leaf papillae. Thus, it seems that resistance to this biotrophic fungus was at least partially dependent on ABA-mediated changes.

Qi et al. [191] presented results about role of ABA during the interaction between wheat plants and the *Fusarium graminearum* fungus. It is worth noting that *F. graminearum* establishes a brief biotrophic relationship with infested plants before switching to the necrotrophic stage. Inoculation of heads of the susceptible cultivar of *T*. *aestivum* with *F*. *graminearum* led to significantly enhanced levels of ABA and its related metabolites, such as DPA, PA, and 7′-hydroxyl-ABA, at 4 days post-inoculation (dpi). Interestingly, mycelia of *F*. *graminearum* were also able to synthesize low amounts of ABA, DPA, and PA. Similar results were obtained by Spence et al. [192] for *M. oryzae,* where it has been presented that mycelia and spores of *M. oryzae* also have the ability to produce ABA. Therefore, the results of Spence et al. [192] and Qi et al. [191] suggest that ABA can be a fungal effector during pathogenesis. This issue was meticulously discussed in the review article by Lievens et al. [193] showing the role of pathogen-origin ABA in suppression of plant defense responses. Qi et al. [191] also checked the effects of exogenous ABA application on fusarium head blight (FHB) progression in wheat plants and found that ABA treatment significantly increased the development of FHB symptoms. In addition, Buhrow et al. [194] also conducted experiments on the interaction between wheat and *F*. *graminearum*. They showed that susceptible and resistant cultivars have different responses regarding ABA. An increased amount of ABA was found in spikes of an FHB-resistant wheat cultivar infected with *F*. *graminearum*, and PA and total ABA catabolites were found to be increased in an FHB-susceptible one, suggesting that ABA inactivation may in fact be a sensitivity factor for this pathogen infection. On the other hand, the application of ABA enhanced the rate of FHB symptom development, especially from symptomatic tissues to neighboring asymptomatic ones. Based on the results of Qi et al. [191] and Buhrow et al. [194], a conclusion can be formulated that the exogenous treatment of ABA enhances susceptibility of wheat to *F*. *graminearum* infection and the spread of FHB symptoms. However, molecular mechanisms of ABA modulation of defense responses against pathogens remain sill elusive. Results by Gordon et al. [3] provide some helpful evidence, as a family of ABA receptors in wheat was identified. The role of the Ta_PYL4AS_A wheat receptor (and its close homologs) in modulating susceptibility to *F*. *graminearum* infection was presented; furthermore, knockdown of Ta_PYL4AS_A and other close homologs in *T*. *aestivum* led to improved resistance to FHB. Therefore, it is probable that not only the level of ABA but also the plant sensitivity to this phytohormone impacts plant resistance to fungi, meaning that even plants showing little to no changes in ABA level (e.g., wheat in response to *M. oryzae*) may in fact differ in ABA-mediated response to biotic stress.

Another common wheat-attacking pathogen is *Tilletia caries.* It is a basidiomycete biotrophic fungus causing common bunt disease in cereals. Maksimov et al. [195] evaluated the phytohormonal response of two wheat species genotypes contrasting in susceptibility to *T. caries* infection. In their experiment, wheat seeds were inoculated with *T. caries* spores and the ABA level was estimated in seedlings at different dpi. In the susceptible plants, enhanced ABA amount was kept for a longer duration, which according to these authors could operate as a factor of virulence. Simultaneously, in the resistant genotype, an increase in ABA level was short-lived and most likely served to trigger defense responses of infected plants. Therefore, a short spike in ABA level seems to be necessary for activation of proper plant response, but longer accumulation of this phytohormone has a negative effect on the resistance to the spread of *T. caries*.

Another disease that often occurs in cereals is wheat leaf rust (WLR). It is a fungal wheat and barley disease caused by species from the *Puccinia* genus, including *P*. *triticina* (bringing brown rust) and *P*. *striiformis* (bringing yellow rust). Li et al. [196] analyzed the gene and protein expression of TaLr35PR5, a wheat pathogenesis-related protein-5, in response to infestation by *P*. *triticina*. Pathogenesis-related proteins-5belong to the family of proteins also known as thaumatin-like proteins [197]. The gene expression of TaLr35PR5 in wheat leaves treated with ABA was rapidly enhanced at 12 h post-application, and afterwards its transcript level decreased. *TaLr35PR5* expression in wheat leaves pretreated with ABA and then inoculated with *P*. *triticina* was significantly stimulated at 24 h post-inoculation (hpi). Li et al. [196] concluded that the gene expression of TaLr35PR5 is induced by ABA and proposed that TaLr35PR5 participates in wheat defense response against *P*. *triticina* infection. The expression of another gene that also belongs to the pathogenesis-related proteins-5 family was identified and analyzed by Zhang et al. [198] Expression analysis and functional characterization of *TaLr19TLP1* from wheat were performed after infection with *P*. *triticina*. The *TaLr19TLP1* mRNA level in wheat leaves pretreated with ABA and next inoculated with *P*. *triticina* was significantly enhanced at 1, 3, 5, and 10 dpi. Therefore, the results presented by Li et al. [196] and Zhang et al. [198] clearly and emphatically suggest that pathogenesis-related proteins-5, including TaLr35PR5 and TaLr19TLP1,also play a significant role in wheat resistance to *P*. *triticina* via the ABA signaling pathway. In addition, transcriptomic data presented by Dobon et al. [199] indicate the validity of ABA-dependent defense signaling in wheat infested with *Puccinia striiformis* f. sp. *tritici* at 1 and 11 dpi.

### 4.3. ABA in Other Diseases

Apart from fungal infection, *T. aestivum* and *H. vulgare* are also exposed to a number of other diseases resulting from viral and bacterial infections, as well as pest attacks. Moreover, pests often act as vectors in spreading viral infections. These factors also result in an increase of the ABA content in plants, and its accumulation is often related to plant sensitivity to a particular disease. As described previously for the participation of ABA in the abiotic stress response, abscisic acid leads to overproduction of ROS, inhibition of plant growth, leaf senescence, and alternations in phloem and xylem flow by changes in intake and conduction of water. All these alternations may play an important role in plant resistance to infection and infestation, either by aggravating the negative consequences of stress or by initiating the systemic response to counteract the infection [200,201].

As previously stated, viral infections also cause changes in ABA signaling in infected *T. aestivum* plants. For example, this was proven by Davis et al. [202] in experiments performed on *T. aestivum* inoculated with BYDV. ABA content was found to increase consistently over the course of the observation period (from 8, through 16 and 24, to 32 dpi) in wheat leaves infected with BYDV. Furthermore, Paulmann et al. [203] demonstrated the increase in ABA content in leaves of a BYDV-susceptible cultivar of *H*. *vulgare* inoculated with BYDV at 6 weeks post-infection in comparison to the noninoculated ones. Interestingly, this increase was not detected in BYDV-infected leaves of BYDV-tolerant barley cultivar. This leads to conclusion that the reduced growth of plants of the susceptible barley cultivar resulted from accumulated ABA molecules, which lead to increased ROS amount, decreased vascular development, and suppressed electrophysiological conductivity of the phloem. Therefore, as a consequence of the high accumulation of ABA leading to damages caused by oxidative imbalance and disturbance of water management, plant susceptibility to virus infection was induced. In addition, Xie et al. [204] published results on defense responses of wheat plants colonized by the English grain aphid (*Sitobion avenae*), the preponderant and devastating pest of wheat and barley which also acts as a BYDV vector [201]. *S. avenae* infestation significantly enhanced the ABA amount in wheat leaves at 24 hpi in comparison to noninfested plants [204]. Moreover, the feeding of another aphid species, the Russian wheat aphid (*Diura phisnoxia*),on barley leaves caused the induction of expression of the *NCED* gene [205], which is associated with ABA synthesis, suggesting that accumulation of abscisic acid may be a common response to aphid infestation.

It should be mentioned that infection with bacterial plant pathogens can also induce ABA accumulation and that the application of this phytohormone can influence the plant response to bacteria-related diseases. For example, *Pseudomonas syringae* pv. *japonica* was shown to induce ABA synthesis in barley plants. Moreover, the increase in ABA was noticed mainly in infected leaves. Furthermore, local application of abscisic acid on barley leaves triggered plant systemic immunity to *Xanthomonas translucens* pv. *cerealis* infection [200]. In the light of these findings, it seems that, in contrast to viral infection, ABA triggers plant resistance to diseases caused by bacteria and is a key element for systemic immunity activation.

## 5. Conclusions

In conclusion, the sensitivity of cereals to adverse environmental factors is mostly determined in the literature on the basis of germination ability and yield, as their main purpose is food production. ABA can affect those parameters. Under abiotic stress, in order to enable plant survival, ABA leads to preservation of water by stomata closure, minimization of transpiration by wax-layer thickening, and accumulation of osmoprotectants and defense-related proteins such as LEA. On the other hand, ABA can accelerate leaf senescence, lower grain numbers and filling rate, and induce seed dormancy. The intensity of these processes is dependent on ABA content; therefore, in some cases, overaccumulation of ABA may in fact intensify the negative effects of applied stresses, which is highly undesirable in agriculture. Furthermore, the presented findings show that ABA can act as a susceptibility factor for diseases caused by wheat and barley pathogens. However, its involvement in the signaling of defense responses against pathogens or pests in wheat and barley is also visible. Based on the research presented in this review regarding the participation of ABA in the response of cereals to abiotic and biotic stresses, it cannot be clearly stated whether ABA is an enemy or savior. In summary, the role of ABA depends on the concentration of this compound and the type of applied stress. Contrary to previous views, the increase of the ABA concentration is not always synonymous with the activation of adequate mechanisms to protect the plant from stress or its consequences. Particular attention should be paid to understanding the importance of ABA participation in response to biotic stresses, because this issue is not sufficiently recognized and seems to be significant for determining future directions in cereal breeding programs.

## Figures and Tables

**Table 1:** Contribution of genes encoding enzymes involved in ABA metabolism in response to abiotic stresses in cereals.

Gene	Species	Type of Manipulation	Effect	Reference
*OsABA1* (*ZEP*)	Rice	Rice *Osaba1* mutant	Wilty phenotype; low ABA content even upon drought	[7]
*TaNCED1*	Wheat	Overexpression of *TaNCED1* in tobacco	Improved drought tolerance; increased ABA content; higher rate of relative water and soluble sugars content	[26]
*HvNCED1*	Barley	Transgenic barley line with downregulation of endogenous *HvNCED1*	Reduced ABA level during prolonged drought stress	[27]
*OsNCED3*	Rice	Overexpression of *OsNCED3* in *A. thaliana*	Improved drought tolerance; increased ABA content; reduced relative water loss	[28]
*TaABA8′OH1*	Wheat	Overexpression of *TaABA8′OH1* in rice	Reduced anther ABA content under cold stress	[29]
*HvABA8′OH1*	Barley	RNAi silencing of *HvABA8′OH1* in barley	Improved drought tolerance; better water use efficiency	[27]
*OsABA8ox1* (*ABA-8′-OH1*)	Rice	Overexpression of *OsABA8ox1* in rice	Reduced drought and cold tolerance due to low ABA content	[30]
